# The Acute Effects of Exercise and Temperature on Regional mtDNA

**DOI:** 10.3390/ijerph18126382

**Published:** 2021-06-12

**Authors:** Mark L. McGlynn, Halee Schnitzler, Robert Shute, Brent Ruby, Dustin Slivka

**Affiliations:** 1 School of Health and Kinesiology, University of Nebraska at Omaha, Omaha, NE 68182, USA; markmcglynn@unomaha.edu (M.L.M.); hmkeller@unomaha.edu (H.S.); rs6qz@virginia.edu (R.S.); 2 School of Integrative Physiology and Athletic Training, University of Montana, Missoula, MT 59812, USA; brent.ruby@mso.umt.edu

**Keywords:** mitochondria, thermoregulation, mtDNA, exercise, copy number, ambient temperature

## Abstract

A reduced mitochondrial DNA (mtDNA) copy number, the ratio of mitochondrial DNA to genomic DNA (mtDNA:gDNA), has been linked with dysfunctional mitochondria. Exercise can acutely induce mtDNA damage manifested as a reduced copy number. However, the influence of a paired (exercise and temperature) intervention on regional mtDNA (MINor Arc and MAJor Arc) are unknown. Thus, the purpose of this study was to determine the acute effects of exercise in cold (7 °C), room temperature (20 °C), and hot (33 °C) ambient temperatures, on regional mitochondrial copy number (MINcn and MAJcn). Thirty-four participants (24.4 ± 5.1 yrs, 87.1 ± 22.1 kg, 22.3 ± 8.5 %BF, and 3.20 ± 0.59 L·min^−1^ VO_2_peak) cycled for 1 h (261.1 ± 22.1 W) in either 7 °C, 20 °C, or 33 °C ambient conditions. Muscle biopsy samples were collected from the vastus lateralis to determine mtDNA regional copy numbers via RT-qPCR. mtDNA is sensitive to the stressors of exercise post-exercise (MIN fold change, −1.50 ± 0.11; MAJ fold change, −1.70 ± 0.12) and 4-h post-exercise (MIN fold change, −0.82 ± 0.13; MAJ fold change, −1.54 ± 0.11). The MAJ Arc seems to be more sensitive to heat, showing a temperature-trend (*p* = 0.056) for a reduced regional copy number ratio after exercise in the heat (fold change −2.81 ± 0.11; *p* = 0.019). These results expand upon our current knowledge of the influence of temperature and exercise on the acute remodeling of regional mtDNA.

## 1. Introduction

The ratio of mitochondrial DNA (mtDNA) to genomic DNA (gDNA), i.e., mtDNA copy number (mtDNA/gDNA), has been widely used to quantify mitochondria found within tissue [1,2,3,4,5,6]. However, others suggest the need for improved measurement standards [7,8]. When mtDNA is damaged, unlike genomic DNA (gDNA), errors are often left unrepaired and can persist within the mitochondria. These persisting mtDNA errors, potentially due to increased reactive oxygen species (ROS), have been associated with mitochondrial dysfunction linked to the aging process and many common diseases, e.g., Alzheimer’s disease, diabetes mellitus, and cancer [1,2,3,4,6]. Additionally, the mtDNA plasmid can be broken down into two regions, the Minor Arc (MIN) and the Major Arc (MAJ). The locations of these regions and the unique steps of mtDNA transcription place the MAJ at greater risk for transcriptional errors [9]. Further, the MAJ region has been highly linked with diseases associated with dysfunctional mitochondria presenting as a reduced MAJ copy number (MAJcn). 

Mitochondria are highly concentrated in skeletal muscle and can undergo damage when the ATP demand is elevated (e.g., a stressor like exercise and/or temperature) via increased production of ROS. Gene expression data has suggested that exercise, when paired with cold and hot ambient temperatures, may have different transcriptional influences [10,11,12,13,14,15,16,17]. More specifically, the pairing of exercise and cold ambient temperature may act in a mitochondrial-enhancing manner through the upregulation of the gene *PGC1α* and its mitochondrial biogenesis-related downstream targets [10,11,15,16]. Whereas exercise in hot ambient conditions may blunt these same mitochondrial-related benefits [10,11,12,17]. However, the exact mechanisms for these altered gene expression results are unclear. Interestingly, acute exercise, usually associated with an enhanced mitochondrial complex (via enhanced content and function), has also demonstrated its potential to induce a reduced mtDNA copy number [18,19]. 

Therefore, public health research has established a link between a reduced MAJcn and an increased risk for disease processes [1,2,3,4]. Whereas it is less clear if there are selective mtDNA regional (MIN vs. MAJ) effects with exercise. Of the limited investigations demonstrating a reduced mtDNA copy number post-exercise, few have adequately controlled their interventions with room temperature or cold ambient conditions [18,19] and, to our knowledge, none have explored the influence of exercise on regional mtDNA copy number. Indeed, the effects of environmental temperature combined with exercise on regional mtDNA copy number are unknown. Therefore, the purpose of this investigation was to determine the acute effects of exercise in cold (7 °C), room temperature (20 °C), and hot (33 °C) ambient temperatures, on regional mitochondrial copy number (MINcn and MAJcn).

## 2. Materials and Methods

### 2.1. Subjects and Study Design

Thirty-six males volunteered and completed the informed consent process for inclusion before they participated in the study. The study was conducted in accordance with the Declaration of Helsinki, and the protocol was approved by the University of Nebraska Medical Center Institutional Review Board. All subjects were considered untrained, that is, they had not participated in any structured cardiovascular exercise program for the last 3 months. Under the American College of Sports Medicine stratification, all participants were considered “low risk” for cardiovascular disease-related events. Samples and descriptive data used in this study are a sub-set obtained from a larger project focused on exercise and temperature acclimation over a longer training period [16,17]. Two participants were removed from analysis due to extreme variation in reference genes and thus the inability to meet the assumption of reference gene stability. The remaining 34 participants were randomly assigned to one of three groups, cold (7 °C, *n* = 12), room temperature (20 °C, *n* = 11), and hot (33 °C, *n* = 11). To minimize the influence of acclimation, data collection was strategically planned. The 7 °C trials were completed during the warmer months (July–August), 20 °C trials were conducted during more temperate months (April–May), and 33 °C trials were conducted during the colder months (January and February). The 3 groups performed initial testing and one experimental cycling trial within their respective temperatures during which thermoregulatory data (heart rate, skin temperature, and core temperature) and 3 muscle biopsies (Pre, Post, and 4hPost) were collected.

### 2.2. Initial Testing

Initial testing of all subjects was completed in standard room temperature conditions and consisted of measuring height (standard standiometer, Seca, Chino, CA, USA) weight (digital output scale, Befour, PS-660, Saukville, WI, USA), body composition (electronic load cell system, Exertech, Dresbach, MN, USA), and maximal aerobic capacity (VO_2_peak). Subjects were instructed to refrain from any strenuous exercise within the 48 h prior to the trial date and to arrive at the laboratory in an overnight-fasted state, also having not ingested caffeine or alcohol within the past 24 h.

Body density was collected via hydrodensitometry in a custom-designed tank and measured via a hydrodensitometry electronic load cell scale (Exertech, Dresbach, MN, USA). Participants were asked to void prior to testing and instructed to completely submerge themselves while seated on the scale. After a correction for estimated residual lung volume analysis [20], density values were then converted to percent body fat via the Siri equation [21]. 

VO_2_peak data was conducted on an electronically-braked cycle ergometer (Velotron, RacerMate, Seattle, WA, USA) while expired gases were collected via a calibrated metabolic system (ParvoMedics TruOne Metabolic System, Sandy, UT, USA). The VO_2_peak protocol started at 95 W and increased by 35 W, with each 3 min stage, until exhaustion. The workload (W) associated with VO_2_peak (Wpeak) was calculated by multiplying the time spent in the final stage by 35 W (the Watt increase per stage) and then adding this to the workload of the previously complete stage.

### 2.3. Experimental Trial 

Subjects were instructed to refrain from any strenuous exercise within the 48 h prior to the trial date and to arrive at the laboratory in a fasted state. The experimental cycling trial was performed at 50% Wpeak for 60 min and conducted in a temperature- and humidity-controlled (40% relative humidity) chamber (Darwin Chambers Co., St. Louis, MO, USA) within the designated temperatures, either cold (7 °C), room temperature (20 °C), or hot (33 °C). These combinations of temperatures and relative intensity were chosen based upon previous works demonstrating altered mitochondrial gene expression [10,11,12,13,14,15,16,17] and to make sure all participants were able to complete the full trial, specifically those within the 33 °C cycling group. All subjects wore short-sleeved T-shirt and standard cycling shorts to maintain consistency among the groups. Heart rate (bpm), core temperature (°C), and skin temperature (°C) were continuously monitored throughout the exercise trial and then averaged over four time points (i.e., 15 min intervals) and a 5 min averaged resting timepoint. Core temperature was measured via a rectal temperature probe (RET-1, Physitemp Instruments Inc., Clifton, NJ, USA) that was self-inserted 12 cm past the anal sphincters. Skin temperature was calculated as the average of chest and back adhesive skin probes (SST-1, Physitemp Instruments Inc., Clifton, NJ, USA). Temperatures were logged using a thermal SD logger (SDL200, Extech, Nashua, NH, USA). Heart rate was monitored via a standard chest strapped heart rate monitor (V800, Polar Electro, Lake Success, NY, USA). Upon the conclusion of exercise, participants recovered for 4 h, resting in a seated position and were provided a pre-packaged standardized meal consisting of ~730 ± 100 Kcal (~43% carbohydrates, 42% fat and 15% protein).

### 2.4. Muscle Biopsies 

Prior to the start of exercise (Pre), immediately post exercise (Post), and 4 h after the exercise trial (4hPost) muscle biopsies were extracted from the belly of the vastus lateralis from each subject (randomized) using the percutaneous needle biopsy technique with the aid of suction, as described previously [22], and more recently modified for effectiveness [23]. Briefly, lidocaine (2–3 mL of 1%) was injected under the skin surface and around the muscle fascia before a small incision was made and the muscle sample obtained. The incision was treated with antibiotic ointment, closed using steri-strips, dressed, and wrapped with a compression bandage. The Pre, Post, and 4hPost biopsies were collected on the same leg approximately 2 cm proximal to the most recent previous biopsies. Based on previous conclusions [24,25], a 4 h post-exercise, rested and fueled, recovery allows coincides with the peak timeframe for gene expression associated with mitochondrial biogenesis. Muscle samples were immediately cleaned of excess blood or visible connective tissue and placed into Allprotect Tissue Reagent (Qiagen, Valencia, CA, USA). Samples were stored overnight at 4 °C and then transferred to −30 °C for additional analyses. 

### 2.5. Mitochondrial Content

Extraction of DNA and analysis methods have been previously reported [16,17]. Briefly, a 16.2 ± 0.2 mg piece of skeletal muscle was homogenized in 800 uL of TRIzol (Invitrogen, Carlsbad, CA, USA) using an electronic homogenizer (PowerGen 150; Fisher Scientific, Hampton, NH, USA). DNA was precipitated with 100% EtOH and the resulting pellet was washed in a 0.1 M sodium citrate and 10% ethanol solution and incubated for 30 min on a tube rotator. The supernatant was discarded, and the pellet resuspended in 400 uL of 8 mM NaOH and then incubated overnight at 50 °C. The DNA was then centrifuged, transferred, and pH was adjusted with 0.1 M HEPES, and quantified via Nanodrop (ND-1000, Wilmington, DE, USA) and adjusted to contain equal amounts of DNA for each sample (19.7 ng/uL). RT-qPCR was performed with Syber Green Mix (SsoAdvanced Universal SYBR Green Supermix, Bio-Rad, Hercules, CA, USA) and primers adapted from [26]. RT-qPCR was run in triplicate using Agilent Technologies AriaMX Real-Time PCR System (Santa Clara, CA, USA) running 1 cycle at 95 °C for 30 s, and 40 cycles of 95 °C for 5 s followed by 10 s at 60 °C. Primers were designed specific to target DNA region and are presented in Table 1. mtDNA copy number was measured via the ratio of mtDNA for each region (MINcn and MAJcn) to a stable genomic gene, *beta-2-microglobulin (B2M)* using the 2^−ΔΔCt^ equation as previously published [5]. To properly compare the influences of the intervention on each mtDNA region, the change in copy number (relative to Pre) was also calculated. 

### 2.6. Statistical Analyses

One-way ANOVAs were used to determined differences between group anthropometrics (age, height, body mass, body fat %, fat mass, fat free mass, and aerobic fitness). A two-way mixed design 5 × 3 (time × temperature) ANVOA was used to determine differences over time (the influence of exercise) and between groups (the influence of temperature) for the 15 min averaged intervals of exercise for heart rate, core temperatures, and skin temperatures. Due to an above average amount of variance within the raw mtDNA values, log transformation was performed on all mtDNA data. A two-way mixed design 3 × 3 (time × temperature) ANOVA was used to compare group means for mitochondrial copy number for the MIN (MINcn) and MAJ (MAJcn) mitochondrial regions. A mixed model 2 × 3 (time × group) ANOVA was used to compare copy number changes, relative to Pre, for the MIN and MAJ mitochondrial regions. If a significant F-ratio was detected, Fisher’s protected least significant difference was used to determine where differences occurred. SPSS Statistics for Windows (Version 27.0. Armonk, NY, USA: IBM Corp) was used for all statistical analysis. A probability of type I error less than 5% was deemed significant (*p* < 0.05). 

## 3. Results

### 3.1. Descriptive Data

The participants in the three temperature groups (7 °C, 20 °C, and 33 °C) were similar for all descriptive variables (age, *p* = 0.602; height, *p* = 0.482; body mass, *p* = 0.996; body fat %, *p* = 0.923; fat mass, *p* = 0.918; fat free mass, *p* = 0.942; aerobic fitness, *p* = 0.481; and peak power, *p* = 0.455). Descriptive data are reported as mean ± SD, see Table 2.

### 3.2. Exercise Trial Data

**Figure 1:**
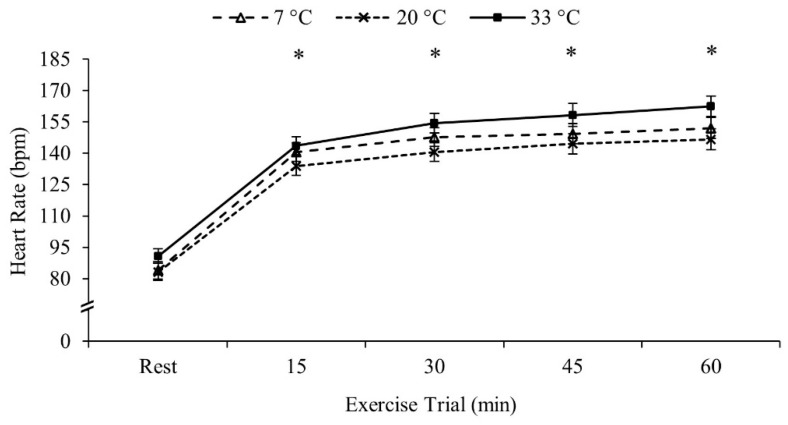
Heart rate data. Heart rate during the exercise trial. Data are reported in mean ± SE. * *p* < 0.05 different from previous time-point.

Heart rate increased throughout the exercise bout and thus all four 15 min intervals were different from each other (Rest < 15 min < 30 min < 45 min < 60 min, *p* < 0.001 for all comparisons). Heart rate was similar between the three temperatures (*p* = 0.211). Heart rate data are presented in Figure 1.

**Figure 2:**
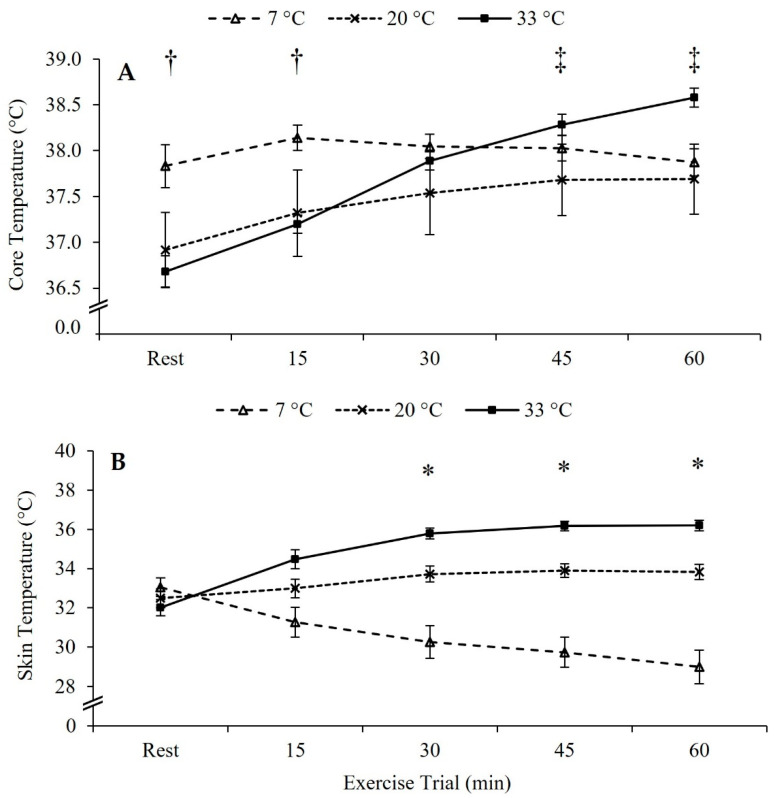
Temperature Data. (**A**) Core temperature during the exercise trial. (**B**) Skin temperature during the exercise trial. Data are reported in mean ± SE. * *p* < 0.05, all groups different. † *p* < 0.05, 7 °C different from other temperatures. ‡ *p* < 0.05, 33 °C different from other temperatures.

Resting core temperature during the initial 5 min of exposure in the cold (7 °C) was greater than room temperature (20 °C; *p* = 0.030) and hot (33 °C; *p* = 0.007). Core temperature remained higher in 7 °C than 20 °C (*p* = 0.048) and 33 °C (*p* = 0.022) through the first 15 min interval; however, groups were similar at 30 (*p* > 0.05) and 45 min (*p* > 0.05). The 33 °C group had a higher core temperature than the 20 °C (*p* = 0.016) and the 7 °C (*p* = 0.044) groups at 60 min. Core temperature data are presented in Figure 2.

Skin temperatures for all groups were similar at rest (*p* > 0.05). Fifteen minutes into the trial, skin temperature within the 33 °C group increased from resting (*p* < 0.001), while the 7 °C group decreased from resting (*p* < 0.001). Consequently, skin temperatures in the 33 °C group were higher than 7 °C group at 15 min into the exercise trial (*p* < 0.001), but no differences were found within the 20 °C (resting, *p* = 0.244; 7 °C*, p* = 0.051; 33 °C, *p* = 0.094). At the 30 min time-point skin temperatures in 7 °C fell below 20 °C (*p* < 0.001) and 33 °C increased above 20 °C (*p* = 0.019). These temperature differences persisted throughout the 60 min exercise trial (*p* < 0.05). Skin temperature data are presented in Figure 2.

### 3.3. Regional mtDNA Quantification

**Figure 3:**
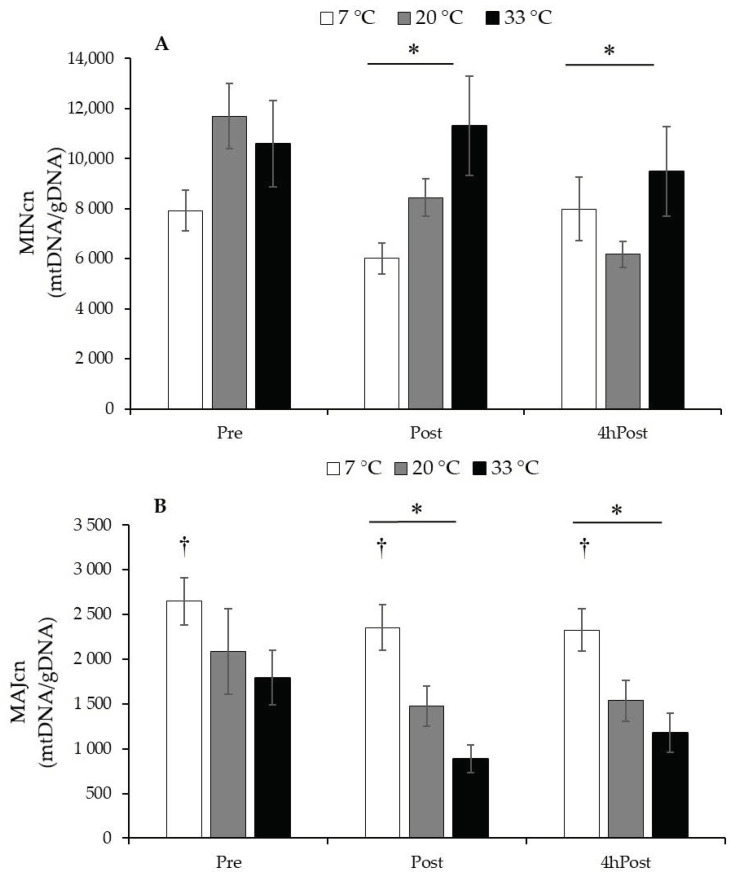
Regional mtDNA copy number. (**A**) mtDNA Minor Arc copy number (MINcn) data relative to reference gene *B2M*. (**B**) mtDNA Major Arc copy number (MAJcn) data relative to reference gene *B2M*. Data are reported in mean ± SE. * *p* < 0.05, less than Pre, main effect over time. † *p* < 0.001, greater than 20 °C and 33 °C, main effect for temperature.

MINcn decreased due to exercise (Post, *p* = 0.002) and were sustained at 4hPost (*p* = 0.009); however, temperature had no influence (*p* = 0.203). MAJcn decreased due to exercise (Post, *p* = 0.001), were sustained at 4hPost (*p* = 0.007), and the 7 °C group had a higher MAJcn than the 20 °C (*p* = 0.012) and the 33 °C groups (*p* < 0.001). mtDNA MINcn and MAJcn data are presented in Figure 3.

**Figure 4:**
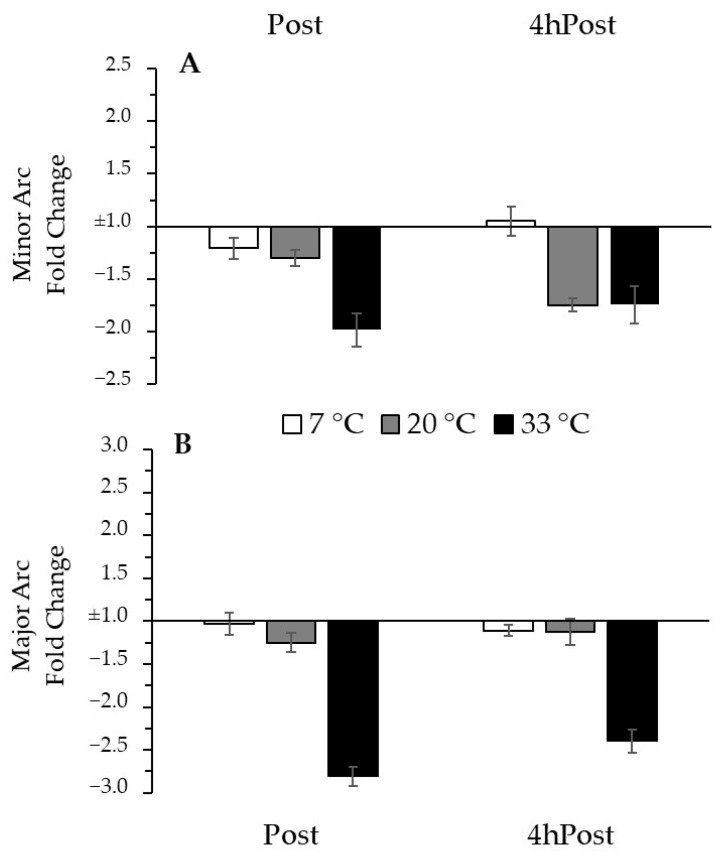
Regional mtDNA copy number change from Pre (**A**) Minor Arc mtDNA change from Pre. (**B**) Major Arc mtDNA change from Pre. Fold change bars present the negative inverse mean change ± SE, relative to (1.0) the reference gene *B2M*.

To adjust for group differences in baseline mtDNA and to focus on the changes that occurred with the intervention, we normalized our data relative to the Pre time-point. Relative to Pre, there was no change in MINcn due to exercise (*p* = 0.518), nor was it influenced by temperature (*p* = 0.114). Relative to Pre, there was no change in MAJcn due to exercise (*p* = 0.149), but there was a trend for an effect of temperature (*p* = 0.056). The trend for a temperature effect would indicate a greater relative decrease with exercise in the 33 °C group compared to the 7 °C group (*p* = 0.019) but no difference compared to the 20 °C group (*p* = 0.467). Thus, after normalization for the initial copy number values, a trend emerged suggesting greater exercise-induced reduction in MAJcn after exercise in the heat. The negative inverse fold change data from Pre for MINcn and MAJcn are presented in Figure 4.

## 4. Discussion

Previous works suggest exercise can induce mtDNA damage manifested as a reduction of mtDNA copy number [7,8,9,18,19,27,28], which is an established marker for mitochondrial dysfunction in many disease processes [1,2,3,4]. Exercising in cold ambient temperatures have demonstrated the potential to upregulate gene expression related to mitochondrial biogenesis [10,11,13,14,15,16]. Whereas, in hot ambient temperatures, there is a post-exercise blunted gene response [10,12,17]. Therefore, the purpose of this investigation was to analyze and compare the acute effects of exercise in different temperature conditions (7 °C, 20 °C, and 33 °C) on mtDNA copy number measured from two distinct mtDNA regions (MIN and MAJ). The main findings of this investigation support previous works [18,19,27,28] suggesting mtDNA is sensitive to the stressors of exercise resulting in the reduction of mtDNA copy number immediately post-exercise and 4 h post-exercise within both mtDNA regions. More importantly, the MAJ region seems to be more sensitive to temperature. After exercise in the cold there were more MAJ copies than in the room temperature and hot conditions, whereas the MIN region was not significantly influenced by temperature. To explore this temperature relationship further, we analyzed the mtDNA change relative to the Pre time-point. This normalization allowed us to remove any initial copy number differences in baseline mtDNA and to focus on the changes due to our exercise and temperature interventions. After this correction, there was still a trend (*p* = 0.056) suggesting that exercise in the cold (7 °C) results in better preservation of mtDNA copy numbers of the MAJ region than when in hot (33 °C) conditions. These data demonstrate a novel finding that mtDNA in the MAJ region are temperature sensitive to the acute effects of exercise and cooler temperatures may attenuate the extent of damage in response to aerobic exercise. 

To our knowledge this is the first acute exercise investigation to compare the regional changes of mtDNA copy number post-exercise within varying temperature conditions. These data suggest a greater loss of MAJ copy numbers after exercise within the heat (33 °C). Theoretically, these reduced copy numbers are based upon the instability of the MAJ because of its unprotected location within the plasmid during the replication processes brought on by the stress of exercise and temperature [9,29]. Consistent reductions in mtDNA copy number have been demonstrated after a bout of moderate or high intensity exercise [18,19,27,28]. However, these previous results did not define the mtDNA region damaged, i.e., the proportions of deletions among the MIN and MAJ. Thus, the novelty of these results suggest cold ambient temperatures may help to preserve the MAJ region. This is of significance because reductions within the MAJ region, calculated via the copy number ratio, have also been strongly associated with development of dysfunctional mitochondria and many disease processes [1,2,3,4]. However, it is a significant leap to suggest that this acute effect may be manifested in chronic alterations or enhanced disease progression. Instead, these findings demonstrate only the acute effects of exercise in different temperatures in a healthy population and within our 4 h post-exercise study window. Nevertheless, there is a potential therapeutic target for a population suspected to have diminished mitochondrial function. These results should be applied to such a population in order to confirm any therapeutic benefits. 

The MAJ region encodes for most of the proteins directly responsible for ATP production via oxidative phosphorylation [9,29] and thus may have profound bioenergetics implications. mtDNA reductions have been previously explained by an increased production of ROS with acute exercise (during and 4 h post-exercise) contributing to increased mtDNA damage [9,18,19,29]. Our laboratory has provided ample evidence of a less than optimal mitochondrial biogenesis adaptive response via mRNA expression when exercising within the heat versus the cold [10,13,17]. The ATP demand of strenuous exercise has demonstrated the potential to induce an imbalance of ROS production [18,19,27,29]. Therefore, the stress of exercise within the heat may, theoretically, compound the stressors upon ATP demand resulting in a greater potential for mtDNA deletions and thus, a reduced copy number. Additional works support the notion that cold ambient temperatures can preferentially stimulate antioxidant components to quench the risk of mtDNA oxidative stress and potentially reduce the magnitude of MAJ copy number deletions [30,31]. Further, both mRNA expression and mtDNA damage, in response to exercise, seem to be improved in cooler temperatures. Taken together, these results may provide a potential explanation for differences in mitochondrial related gene expression results that we have consistently found with our exercise and temperature investigations [10,11,12,13,14,15,16,17]. However, the exact relationship between mtDNA and exercise induced mRNA expression is not clear and the mechanisms for each appear independent. Recent reviews explain the difficulty of our current understanding of the relationships among exercise, ROS, mtDNA, and the health of the mitochondrial complex [29,32]. 

The application of mitochondrial copy number seems to have some limitations. While the quantification of the MAJ region via copy number has been correlated with numerous pathological conditions [1,2,3,4], it has not been applied to these acute timeframes. Normally, mitochondrial copy number is used to provide a rough quantitative value for mitochondria within tissue and an indirect measure of mitochondrial capacity. More importantly, the mtDNA gene targets and the acute timeframe of these methods only indirectly measure mitochondria. In our experimental framework mtDNA damage is the result of the synthetic primers unable to anneal with the mRNA template due to nucleotide deletions among the MIN and MAJ regions. Nevertheless, the current data support previous findings on the acute effects of exercise and provide further information suggesting mtDNA in the MAJ arc is more sensitive to the exercise stresses in the heat. 

Of note, the current data is consistent with previous works that have demonstrated an increased core temperature within cold ambient environments, due to the body preferentially vasoconstricting and conserving metabolic heat generated within the core [10,11,13,14,15,16]. Whereas during exercise, core and skin temperatures reacted as expected (i.e., increased in the heat and decreased in the cold). Indeed, this vascular phenomenon diminished within core temperature (at 30 min) and continually climbed above the other temperature conditions. Additionally, this acute exercise intervention induced similar rises in heart rate and power output, whereas core and skin temperatures differed among the temperature treatments. Previous mitochondrial gene expression data suggested our 4-h recovery timeframe was optimal for mitochondrial biogenesis gene expression [15,24]. However, this may not be the complete timeframe, nor temperature, for optimal mtDNA replication markers as others have found an exercise-induced mtDNA copy number reduction sustained up to 24 h post exercise [18,19]. Furthermore, these findings were an acute subset of a longer three-week training study focused on temperature acclimation [18]. Hence, these data collection times were strategically planned to occur in concert with seasonal weather changes so that subjects were unacclimated to their temperature condition. However, the extent of alterations that occur during a longer recovery time and/or unacclimated conditions are beyond the scope of this initial investigation. This investigation provides evidence to support the temperature sensitivity of mtDNA after exercise and future work should address the time-course and implications on mitochondrial function. 

## 5. Conclusions

Here we report mtDNA is sensitive to the stressors of exercise resulting in the reduction of mtDNA copy number within both (MIN and MAJ) regions. Additionally, greater copy numbers were found within the cold condition. After correcting for pre-exercise copy number values, a trend suggested the MAJ underwent more mtDNA deletions within the heat, which may help to explain the preservation of copy numbers within the cold. This study extends previous reports on mtDNA deletions by better describing the impact of exercise and temperatures upon the mtDNA and its regions [18,19]. These findings help to better describe the relationships of exercise, temperature, and acute mtDNA damage, with implications on the region of mtDNA investigated.

## Figures and Tables

**Table 1:** Probes and primers used for real time qRT-PCR.

Genetic Target	Forward Primer	Reverse Primer
Genomic DNA	GCTGGGTAGCTCTAAACAATGTATTCA	CCATGTACTAACAAATGTCTAAAATGGT
mtMIN Region	CTAAATAGCCCACACGTTCCC	AGAGCTCCCGTGAGTGGTTA
mtMAJ Region	CTGTTCCCCAACCTTTTCCT	CCATGATTGTGAGGGGTAGG

**Table 2:** Group characteristics and exercise trial data.

Characteristics	7 °C (*n* = 12)	20 °C (*n* = 11)	33 °C (*n* = 11)
Age (yrs)	25.6 ± 6.5	23.5 ± 3.9	24.1 ± 4.8
Height (cm)	180.9 ± 7.4	177.9 ± 6.8	177.7 ± 5.6
Body mass (kg)	86.9 ± 20.7	87.6 ± 26.1	86.6 ± 19.6
Body fat (%)	21.7 ± 7.2	23.1 ± 11.1	22.8 ± 7.3
Fat mass (kg)	19.9 ± 10.1	22.1 ± 16.9	20.8 ± 10.6
Fat free mass (kg)	67.1 ± 11.7	65.5 ± 12.9	65.8 ± 10.3
VO_2_peak (L·min^−1^)	3.47 ± 0.56	3.25 ± 0.74	3.17 ± 0.48
Peak Power (Watt_max_)	234.3 ± 34.1	215.7 ± 50.3	216.8 ± 34.6

Data are mean ± SD.

## Data Availability

Please request the datasets from this investigation from the corresponding author.
